# Amygdala Represents Diverse Forms of Intangible Knowledge, That Illuminate Social Processing and Major Clinical Disorders

**DOI:** 10.3389/fnhum.2018.00336

**Published:** 2018-08-22

**Authors:** C. S. E. Weston

**Affiliations:** Independent Researcher, Amboise, France

**Keywords:** amygdala, perirhinal cortex, intangible knowledge, embodied cognition, paradoxical functional facilitation, autism spectrum disorder

## Abstract

Amygdala is an intensively researched brain structure involved in social processing and multiple major clinical disorders, but its functions are not well understood. The functions of a brain structure are best hypothesized on the basis of neuroanatomical connectivity findings, and of behavioral, neuroimaging, neuropsychological and physiological findings. Among the heaviest neuroanatomical interconnections of amygdala are those with perirhinal cortex (PRC), but these are little considered in the theoretical literature. PRC integrates complex, multimodal, meaningful and fine-grained distributed representations of objects and conspecifics. Consistent with this connectivity, amygdala is hypothesized to contribute meaningful and fine-grained representations of intangible knowledge for integration by PRC. Behavioral, neuroimaging, neuropsychological and physiological findings further support amygdala mediation of a diversity of such representations. These representations include subjective valence, impact, economic value, noxiousness, importance, ingroup membership, social status, popularity, trustworthiness and moral features. Further, the formation of amygdala representations is little understood, and is proposed to be often implemented through embodied cognition mechanisms. The hypothesis builds on earlier work, and makes multiple novel contributions to the literature. It highlights intangible knowledge, which is an influential but insufficiently researched factor in social and other behaviors. It contributes to understanding the heavy but neglected amygdala-PRC interconnections, and the diversity of amygdala-mediated intangible knowledge representations. Amygdala is a social brain region, but it does not represent species-typical social behaviors. A novel proposal to clarify its role is postulated. The hypothesis is also suggested to illuminate amygdala’s involvement in several core symptoms of autism spectrum disorder (ASD). Specifically, novel and testable explanations are proposed for the ASD symptoms of disorganized visual scanpaths, apparent social disinterest, preference for concrete cognition, aspects of the disorder’s heterogeneity, and impairment in some activities of daily living. Together, the presented hypothesis demonstrates substantial explanatory potential in the neuroscience, social and clinical domains.

## Introduction

Amygdala is a complex brain structure, that is located in medial temporal lobe, and comprises some 13 nuclei and cortical areas in monkey (Freese and Amaral, 2009). Major nuclei are the lateral, basal, accessory basal and central amygdaloid nuclei, which constitute 33%, 27%, 10% and 3% of total amygdaloid neuron numbers in human, respectively (Schumann and Amaral, 2005). Amygdala is an ancient structure, with a homolog of it present in reptiles and origins in still earlier animal groups (Laberge et al., 2006; Murray et al., 2009), as well as exceptionally widely interconnected in primate (Young et al., 1994; Freese and Amaral, 2009). Amygdala is also a multi-functional brain region, with some functions being well established, such as emotional memory enhancement (Hamann, 2009; Murty et al., 2010; McIntyre et al., 2012; McGaugh, 2013), and participation in the fear network (Shin and Liberzon, 2010; LeDoux, 2014; Janak and Tye, 2015). Other functions are more recently reported, as with the representation of features of behavioral plans (Hernádi et al., 2015; Zangemeister et al., 2016).

Amygdala is likely involved in further cognitive functions, and has long been suggested to mediate importance, significance, salience and so forth (Geschwind, 1965; Gloor et al., 1982; Amaral and Price, 1984; Sander et al., 2003; LaBar and Warren, 2009; Phelps, 2009; Adolphs, 2010; Pessoa and Adolphs, 2010). These proposals are built upon but reconceptualized and elaborated further in this hypothesis article. Specifically, rather than few, coarse-grained, often genetically pre-specified representations of salience, relevance, or related concepts, the novel hypothesis is presented that a principal function of amygdala is the representation of diverse, meaningful and fine-grained, intangible knowledge representations. Moreover, amygdala often elaborates such knowledge representations through interactions between brain, body and environment, in accordance with the postulates of embodied cognition (Varela et al., 1991; Chiel and Beer, 1997; Clark, 1999, 2008). The hypothesis should significantly advance understanding of amygdala, its involvement in normal social functions, its contribution to clinical disorders with amygdala involvement, such as posttraumatic stress disorder, anxiety disorders, depressive disorders and autism spectrum disorder (ASD; Koenigs et al., 2008; Mayberg, 2009; Schumann and Amaral, 2009; Shin and Liberzon, 2010; Amaral et al., 2011; Price and Drevets, 2012; Weston, 2014), and is relevant for a comprehensive account of amygdala function. Generally, the functions of brain structures are best understood through connectivity findings, and through behavioral, physiological and related findings (Behrens and Johansen-Berg, 2005; Passingham and Wise, 2012), so such findings pertaining to amygdala and related regions will be examined in the next two sections, respectively.

## Connectivity

### Amygdala Connectivity

Connectivity is largely elucidated through neuroanatomical tracing studies in monkey and rodent, as well as physiological studies. Amygdala interconnections are broadly similar in rodent and non-human primate, and likely in human also (McDonald, 1998). Amygdala receives important inputs from the great majority of sensory modalities, and these relay via high-level subregions of sensory processing pathways. Visual inputs from the anterior-most part of the ventral visual object pathway, principally area TEa in ventral temporal cortex (VTC) in monkey, relay to amygdala (Iwai and Yukie, 1987; McDonald, 1998; Stefanacci and Amaral, 2000, 2002; Freese and Amaral, 2009). Biological motion and multimodal information are processed in superior temporal sulcus (STS; Beauchamp, 2005; Pelphrey et al., 2005; Saygin, 2007), of which predominantly anterior STS relays to amygdala (McDonald, 1998; Stefanacci and Amaral, 2000, 2002; Freese and Amaral, 2009). These interconnections are further supported by findings that early amygdala lesions in monkey and human result in significant degradation of anterior subregions of VTC and STS (Boes et al., 2012; Grayson et al., 2017). Auditory inputs from the high-level auditory area, in monkey predominantly area TAa located in superior temporal gyrus (STG), relay to amygdala (McDonald, 1998; Stefanacci and Amaral, 2000, 2002; Yukie, 2002; Freese and Amaral, 2009). Olfactory information is gathered by sensory cells of the olfactory epithelium, and projected to the olfactory bulb, primary olfactory cortex, thence to secondary olfactory cortex in orbitofrontal cortex (OFC; Shepherd, 2006; Patin and Pause, 2015). The olfactory bulb and olfactory cortices project to several amygdaloid nuclei (McDonald, 1998; Pitkänen, 2000; Patin and Pause, 2015). Further, neuroimaging studies of healthy humans have found reliable amygdala involvement in odor processing (see for review, Patin and Pause, 2015).

Somatosensory, gustatory, nociceptive and viscerosensory information are collected by specialized receptors and processed through multiple and complex sensory processing pathways that include subregions of insula (Friedman et al., 1986; Ostrowsky et al., 2000; Saper, 2000, 2002; Craig, 2002; Gauriau and Bernard, 2002; Pritchard and Norgren, 2004; Mazzola et al., 2006; Small, 2010). Somatosensory information relays from posterior insula to amygdala (Friedman et al., 1986; McDonald, 1998; Pitkänen, 2000), and multiple forms of somatosensation are thereby relayed to amygdala. These include, as found by single-cell recording in monkey, tactile stimulation of the face, for stimuli in the mouth the features of viscosity, fatty texture, grittiness, irritation (elicited by capsaicin) and temperature (Kadohisa et al., 2005; Mosher et al., 2016), and as found by fMRI in human, reducing bodily temperature sensations (Oi et al., 2017). Gustatory information relays from anterior insula to amygdala (McDonald, 1998; Pitkänen, 2000), and single-cell recording studies in monkey report gustatory processing in amygdala (Kadohisa et al., 2005). Nociceptive information relays from posterior insula to amygdala (McDonald, 1998; Gauriau and Bernard, 2002). In addition, clinical and experimental neuroimaging studies of nociceptive processing in human, report activation of a network of brain regions that includes amygdala (see for meta-analysis, Simons et al., 2014).

Viscerosensory nerves of the autonomic nervous system (ANS) collect diverse forms of information from widespread bodily systems, including heart, lungs, gut, pelvic organs and so forth, and such information is processed through multiple pathways, that include the insula (Loewy, 1990; Saper, 2002). Subregions of insula relay viscerosensory information to amygdala (McDonald, 1998; Pitkänen, 2000). Consistent with this neuroanatomy, in human respiratory challenge activates a network of brain regions that includes amygdala (Brannan et al., 2001; Liotti et al., 2001; Evans et al., 2002; von Leupoldt et al., 2009). In physiological studies with animals, manipulations of blood pressure, and of blood acidity, have been found to modulate amygdala neuronal activity (Knuepfer et al., 1995; Ziemann et al., 2009). The stress hormone epinephrine binds to vagus neurons of the parasympathetic subdivision of ANS, which projects via subcortical and cortical routes to amygdala (Schreurs et al., 1986; Roozendaal et al., 1997; Saper, 2002; McGaugh, 2004, 2013). Another stress hormone, cortisol, binds directly to glucocorticoid receptors on amygdala, predominantly the basal amygdaloid nucleus, and to glucocorticoid receptors on Nucleus of the Solitary Tract (NTS) neurons in brainstem which project to amygdala (Roozendaal et al., 1997; Saper, 2002; McGaugh, 2004, 2013).

Perirhinal cortex (PRC) comprises Brodmann’s areas (BAs) 35, 36, and in the temporal pole part of 38 (also named area TG by von Bonin and Bailey, 1947; Suzuki and Amaral, 1994; Davies et al., 2004). Heavy inputs from PRC originate extensively but most densely from polar PRC, and relay extensively to amygdala, most heavily to lateral, basal, and accessory basal nuclei (Stefanacci et al., 1996; Stefanacci and Amaral, 2000). Lateral, basal, accessory basal and periamygdaloid cortex return the heaviest projections, and relay most densely to polar regions of PRC (Amaral and Price, 1984; Stefanacci et al., 1996). Inputs specifically to lateral amygdaloid nucleus in monkey have been traced quantitatively, and the findings were that the heaviest unimodal sensory input is received from high-level visual area TE in VTC. The heaviest input of all to lateral amygdaloid nucleus, at over twice the magnitude of TE input, is received from PRC (Stefanacci and Amaral, 2000). Similarly for reciprocal projections, (Amaral and Price, 1984, p. 492) report that of amygdala projections to temporal and occipital lobes “the heaviest projections are to the temporal pole (especially area TG) and the inferior temporal cortex (especially areas 35, 36).” Furthermore, neonatal amygdala lesions in monkey produced the heaviest brain structural degradation in PRC (Grayson et al., 2017).

Inputs of modest and robust magnitude relay to amygdala from parahippocampal cortex (PHC) and entorhinal cortex (ERC), respectively (Stefanacci et al., 1996; Suzuki, 1996; Stefanacci and Amaral, 2000; Freese and Amaral, 2009), and are reciprocated modestly to PHC and heavily to ERC (Amaral and Price, 1984; Stefanacci et al., 1996). Further, moderate to robust inputs are received by amygdala from OFC and anterior cingulate cortex (ACC), and heavy return projections relay to OFC and ACC (Amaral and Price, 1984; Stefanacci and Amaral, 2002; Freese and Amaral, 2009; Vogt, 2009). Amygdala also receives and relays dense interconnections with BA 45 in ventrolateral prefrontal cortex (VLPFC; Gerbella et al., 2014). Numerous subcortical projections that are mainly reciprocal also interconnect with amygdala, largely with thalamus, hippocampus, multiple brainstem regions, hypothalamus, striatum and basal forebrain (Davis, 1992; Suzuki, 1996; Pitkänen, 2000; Freese and Amaral, 2009).

Taken together, the great majority of sensory systems project heavily to robustly to amygdala, and their high-level subregions relay most frequently and strongly to lateral, basal and central amygdaloid nuclei; in addition, amygdala returns heavy projections to extensive parts of visual cortex, STS, STG and insula (Amaral and Price, 1984; Yukie, 2002; Freese and Amaral, 2005, 2006, 2009). There are generally robust to heavy amygdala interconnections with multiple further cortical areas, and with subcortical brain regions. PRC relays heavily and reciprocally to lateral, basal and accessory basal nuclei, and these are likely the heaviest amygdala interconnections of all; this suggests they mediate a principal amygdala function.

### Perirhinal Cortex

**Figure 1:**
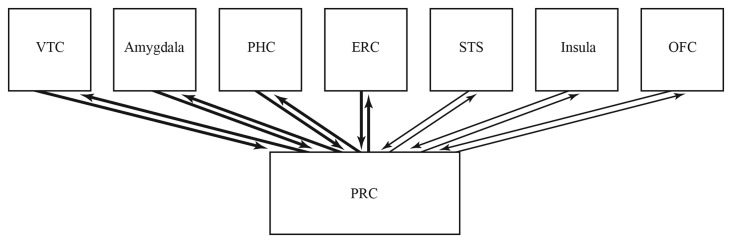
Summary of the hypothesis. PRC integrates complex, multimodal, meaningful and fine-grained, distributed representations. Amygdala interconnects especially heavily and reciprocally with PRC, suggesting it contributes specialized representations for integration by PRC. Consistent with this, amygala represents such forms of intangible knowledge as valence, economic value, importance, noxiousness, social status, trustworthiness and social popularity. Taken together, it is hypothesized that amygala represents diverse forms of intangiable knowledge, that participate in distributed represntations of humans, objects and other stimuli. *VTC*, ventral temporal cortex; *PHC*, parahippocampal cortex; *ERC*, entorhined cortex; *STS*, superior temporal sulcus; *OFC*, orbitofrontal cortex; *PRC*, perirhined cortex.

PRC integrates complex, multimodal, meaningful and fine-grained, distributed object representations (Taylor et al., 2006, 2007, 2009; Cowell et al., 2010; Clarke et al., 2013; Mundy et al., 2013; Behrmann et al., 2016). Further, neural interconnections that are heavy and reciprocal support recurrent processing, that crucially facilitates the processing and development of meaningful, and fine-grained representations (Rossion et al., 2003, 2012; Hegdé, 2008; Cardin et al., 2011; Naci et al., 2012; Clarke et al., 2013; Kravitz et al., 2013; Martin, 2016; Sato et al., 2017a). Together, PRC’s function, and the heavy and reciprocal interconnections of amygdala and PRC, suggest that amygdala likewise contributes meaningful and fine-grained representations for integration by PRC (Figure 1).

Complex, multimodal, and meaningful, distributed representations comprise sensory, motor and other specialized components that are distributed across the primate brain, and this is supported by neuroimaging studies and neuropsychological findings. For instance, faces and other complex objects are represented across multiple brain regions, the chief ones including inferior occipital cortex (IOC), fusiform gyrus (FG) and other subregions of VTC, STS, amygdala, insula, OFC and temporal pole (Allport, 1985; Rossion et al., 2003; Bouvier and Engel, 2006; Atkinson and Adolphs, 2011; Ku et al., 2011; Perrodin et al., 2015). Such distributed representations are integrated by means of nodes or hubs, which also efficiently reactivate those networks so as to achieve knowledge reactivation and retrieval (Barsalou et al., 2003; Barsalou, 2008; Martin, 2009; Meyer and Damasio, 2009). A major such hub is PRC (Taylor et al., 2006, 2007, 2009; Cowell et al., 2010; Clarke et al., 2013; Mundy et al., 2013; Behrmann et al., 2016). PRC’s function of integrating complex, multimodal, meaningful and fine-grained, distributed object representations, is supported by neuropsychological and neuroimaging studies that place demands on such functions.

Human neuropsychological and monkey lesion studies report that lesions to PRC produce visual recognition and visual discrimination impairments for complex object stimuli, but not for more basic ones (Lee et al., 2005, 2006; Saksida et al., 2007; Taylor et al., 2009; Cowell et al., 2010; Hoffman et al., 2014; Behrmann et al., 2016). Neuroimaging studies have examined brain activations during tasks of naming the presented pictures of common objects. The findings were that the task of general naming (e.g., animal, musical instrument) that requires coarse-grained representations, engaged posterior VTC regions, whereas the task of specific naming of the same objects (e.g., zebra, saxophone) that requires complex fine-grained representations, engaged these and additional regions including PRC (Tyler et al., 2004; Clarke and Tyler, 2014). Studies using related paradigms have reported concordant findings (Mundy et al., 2013; Abel et al., 2015; Mollo et al., 2017). Furthermore, temporal pole, PRC and adjacent anterior brain regions participate in recurrent processing with posterior brain regions during cognitive processing. This is evidenced by electroencephalographic (EEG) and magnetoencephalographic (MEG) neuroimaging, as well as transcranial magnetic stimulation (TMS) manipulations, during the performance of challenging object processing tasks (Naci et al., 2012; Chiou and Lambon Ralph, 2016; Mollo et al., 2017). Thus, convergent evidence supports meaningful and fine-grained representations being processed and integrated in PRC.

The formation of persisting knowledge representations results from consolidation processes, whereby initially transient neural activity is transformed into persisting neural representations (Brashers-Krug et al., 1996; Karni et al., 1998; Maquet, 2001). There is abundant and convergent evidence from rodent studies that consolidation is implemented in amygdala (Li et al., 2013; Cestari et al., 2014; LeDoux, 2014; Janak and Tye, 2015; Fanselow and Wassum, 2016; Schiff et al., 2017), including consolidation in the amygdala-PRC circuit (Perugini et al., 2012; Laing and Bashir, 2014). In addition, a study of seven surgical patients with intracranial electrodes implanted in amygdala, presented the patients with degraded fearful and happy faces, and their task was to judge the faces as either fearful or happy. It was found that neuronal activity of a subset of amygdala neurons encoded the patients’ subjective judgments, rather than the physical features, of the face stimuli (Wang et al., 2014). Thus, amygdala encodes representations that are persisting and early evidence suggests likely meaningful too.

Taken together, knowledge representations are distributed across multiple specialized brain regions. Meaningful, fine-grained visual object representations, as well as high-level representations in other modalities, are processed and integrated into complex, multimodal, meaningful and fine-grained, distributed object representations by PRC (Taylor et al., 2006, 2007, 2009; Cowell et al., 2010; Clarke et al., 2013; Mundy et al., 2013; Behrmann et al., 2016). The interconnections of amygdala and PRC, which are especially heavy as well as reciprocal, amygdala implementation of consolidation processes, and early intracranial recording findings consistent with meaningful amygdala representations, together suggest that amygdala likewise contributes meaningful and fine-grained representations for integration by PRC. These representations are hypothesized to concern intangible knowledge. Intangible knowledge is an important component of cognition (see later section: significance for social processing), but it has been little researched as evidenced by few entries in the Medline database. There is no accepted definition, so it is provisionally defined as non-physical or non-concrete features of stimuli, that ubiquitously and substantively modulate behavior, cognition, and emotion. Likewise, the formation of intangible knowledge representations is little researched, but some forms of it are likely self-generated, often through interactions between body, brain and environment. In the next section, these cognitive hypotheses are supported with behavioral, neuroimaging, neuropsychological and physiological evidence, for amygdala’s representation of diverse forms of intangible knowledge.

## Behavioral, Neuroimaging, Neuropsychological and Physiological Evidence

### Subjective Valence Representation

In a study with rhesus monkeys, individual abstract visual images were paired with liquid reward, or aversive air-puffs. The monkeys successfully learned the valence (positive or negative) of each image, and this was expressed by behavioral responses of anticipatory licking or blinking, respectively. After training, the valences were reversed, so that images formerly paired with reward were now paired with air-puffs, and vice versa. Single-cell recording of amygdala neurons revealed that amygdala neuronal activity predominantly encoded image valence, and to a limited extent, image identity. Moreover, after the valence reversals, amygdala neuronal activity was rapidly modified to again reflect image valence (Paton et al., 2006). A subsequent single-cell recording study with rodents, reported that amygdala neurons preferentially encode positive or negative valence (consistent with Paton et al., 2006), and further that this is related to their anatomical projection targets (Beyeler et al., 2016).

A large literature has developed on sensory-specific satiety and its effects on the subjective valence of foods. Specifically, human or monkey subjects are allowed to eat a food until satiety. They are then presented with a choice of that food and another one, and their preference for the first food has commonly fallen relative to the pre-feeding baseline condition. That is, the subjective valence of that food falls during satiety (O’Doherty et al., 2000; Small et al., 2001; Murray and Rudebeck, 2013). Extensive investigations of the brain network underlying this effect indicate that amygdala, OFC and mediodorsal thalamus are involved (Machado and Bachevalier, 2007; Rudebeck and Murray, 2011; Murray and Rudebeck, 2013). Moreover, combining the sensory-specific satiety paradigm with transient inactivation of amygdala in monkey, revealed that amygdala generates revised valence representations of the satiated food (Wellman et al., 2005). Similarly, a human study using satiety manipulations and fMRI, found that subjective valence (attractiveness) representations of foods were mediated by amygdala (Piech et al., 2009). Such valence representations are relayed to anterior OFC which is likely an integrative hub for high-level multimodal food desirability representations (Kringelbach and Rolls, 2004; Rolls, 2005; Price, 2006; Piech et al., 2009; Murray and Rudebeck, 2013).

Further studies have used musical stimuli varying in subjective valence from unpleasantness to intense pleasantness or joyfulness, combined with fMRI or intracerebral electrode arrays to measure human brain activity. The findings were that amygdala is centrally involved in the network mediating musical valence representation (Koelsch et al., 2013; Koelsch and Skouras, 2014). Further, amygdala generates such representations with a relatively lengthy latency, and it modulates the activity of other major network regions, specifically auditory cortex and OFC (Kumar et al., 2012; Omigie et al., 2015a,b). These findings suggest a principal and high-level role of amygdala in the circuit mediating the subjective unpleasantness-pleasantness representations of music. Collectively, diverse paradigms support the generation and representation of subjective valence, a form of intangible knowledge, in amygdala.

### Impact Representation

Amygdala likely processes the “impact” feature of stimuli. High impact features are those features that make stimuli striking and powerful, and often characterize photographs of trouble spots and crises across the world. In a study of this feature, healthy subjects were presented with photographs of stimuli that were high impact or low impact, as well as control stimuli, and inverted neutral stimuli. The task was to respond by button press to all inverted images, and subjects were neuroimaged with fMRI while they performed it. The findings were that high impact stimuli engaged amygdala, and this effect remained after controlling for such potential confounds as arousal, pleasantness, distinctiveness and visual complexity (Ewbank et al., 2009). Thus, amygdala mediates impact representations, a further form of intangible knowledge, but further replications of this finding are desirable.

### Economic Value Representation

Amygdala participates in planning processes, and it represents the values of the benefits and other features of plans. In a physiological study, monkeys were presented with two cues on each trial. They could freely choose by means of saccades to one cue to consume a fruit juice reward immediately (“spend choices”), or they could choose by saccades to the other cue to defer consumption, and thus accumulate multiple fruit juice rewards (“save choices”), which were further augmented with “interest.” Single cell recording of amygdala neurons during the performance of this task, revealed that separate populations of amygdala neurons proactively represented the economic value and length of plans, and that these were predictive of subsequent actions. Moreover, such representations were reset when a new plan commenced after a “spend choice,” and were absent when plans were not engaged during instructed sequences of trials (Hernádi et al., 2015). The key findings above were replicated in a study using healthy human subjects and fMRI neuroimaging (Zangemeister et al., 2016).

### Noxiousness Representation

The hypothesis that amygdala contributes noxiousness representations, generated from nociceptive experiences, to distributed object representations has not been explicitly examined, but is supported by the behavioral study of Kavaliers et al. (1999). Initially, deer mice have no knowledge of biting stable flies, as they do not attempt to avoid those that have had their mouthparts surgically removed. After having been bitten by intact biting stable flies, however, the deer mice on subsequent encounters burrow and take other actions to avoid the biting stable flies, as well as activate endogenous analgesic processes, as demonstrated by the hotplate test. They engage in the same activities even on encountering stable flies whose mouthparts have been surgically removed, so cannot have bitten them. In contrast, the deer mice do not manifest such behaviors when encountering visually similar but non-biting houseflies (Kavaliers et al., 1999). Thus, deer mice evidently form knowledge representations that specifically relate to stable flies and incorporate their noxious character. It appears likely that the crucial interactions that give rise to the latter representation are the stable flies’ bites and the resulting pain suffered by the deer mice (Kavaliers et al., 1999). Together, environment, body, and brain collaborate to generate specific adaptive knowledge, in accordance with embodied cognition.

Amygdala likely plays a crucial role in these effects for multiple reasons. Fear conditioning experiments, which possess common features with the Kavaliers et al.’s (1999) design but use electric shocks instead, have demonstrated an important contribution for amygdala (LeDoux, 2014; Fanselow and Wassum, 2016; Schiff et al., 2017). Nociceptive information is relayed to amygdala, as summarized earlier, and there is substantive evidence that consolidation of nociceptive activations is implemented in amygdala (Neugebauer et al., 2003; Bird et al., 2005; Ikeda et al., 2007; Veinante et al., 2013; Neugebauer, 2015; Shinohara et al., 2017). In addition, disruption of consolidation in a related study by means of injection of NMDA receptor antagonist into the peritoneum of deer mice, was found to abolish the adaptive behaviors and analgesia (Kavaliers et al., 1999, Experiment 2). These results also parallel those in fear conditioning studies (Fanselow and Kim, 1994; Goosens and Maren, 2004) and are consistent with a substantive role for amygdala. Further, encounters of experienced deer mice with biting stable flies elicited endogenous analgesia, which is known to crucially involve amygdala (Helmstetter et al., 1998; Bingel et al., 2006; Eippert et al., 2009; Rouwette et al., 2012; Veinante et al., 2013; Maire et al., 2016), further supporting its involvement. Additionally, amygdala lesions or inactivation performed in pain experiments have not affected baseline levels of pain sensitivity nor of latency of pain responses (Fox and Sorenson, 1994; Bernard et al., 1995; Manning et al., 2001, 2003; Veinante et al., 2013); amygdala also modulates cognition during pain (Veinante et al., 2013); together these findings support amygdala mediation of high-level pain-related functions. In summary, the nociceptive effects of stable fly bites lead deer mice to generate specific knowledge representations of those insects that include their noxious character, and it is suggested the latter representation comprising intangible knowledge, is mediated by amygdala.

### Importance Representation

Amygdala has long been suggested to represent significance or importance (Geschwind, 1965; Gloor et al., 1982; Amaral and Price, 1984; Sander et al., 2003; LaBar and Warren, 2009; Phelps, 2009; Adolphs, 2010; Pessoa and Adolphs, 2010), and it may generate such representations on embodied cognition principles. A stimulus characterized by importance is one “having much import or significance; carrying with it great or serious consequences; weighty, momentous, grave, significant,” as defined by the Oxford English Dictionary Online (1989). An instance in modern times is that employees regard their employers and their associated powers as important, as employees can experience great consequences (promotion, unemployment) from interactions with their employer. Important stimuli mobilize enhanced arousal, effort, perceptual processing, attention, cognition and other functions in an individual, so that the important stimuli can be met with prepared and proportionate resources.

Amygdala participates in distributed representations (see earlier), and it can drive the above enhanced functions for important stimuli, because it projects to an extensive array of brain regions (Amaral and Price, 1984; Young et al., 1994; McDonald, 1998; Freese and Amaral, 2009). Specifically, amygdala (predominantly the central nucleus) projects heavily to hypothalamus and NTS, projects to an array of brainstem regions, including the reticular formation, periacqueductal gray, laterodorsal tegmental nucleus, ventral tegmental area, locus coeruleus and dorsal motor nucleus of the vagus, and projects heavily and reciprocally to the parabrachial complex (PBC; Davis, 1992; Bernard et al., 1993, 1995; Pitkänen, 2000; Freese and Amaral, 2009). Such projections enable amygdala to enhance arousal and effort through modulation of ANS, hormonal, cardiovascular, respiratory, digestive and other visceral systems.

Amygdala sends heavy and excitatory projections to visual cortical areas (Amaral and Price, 1984; McDonald, 1998; Freese and Amaral, 2005, 2006, 2009) enabling it to enhance visual perceptual processing and attention. Amygdala-driven enhancement of vision and attention is further supported by human neuropsychological and monkey lesion findings, that provocative stimuli that enhanced visual processing in healthy controls, did not do so in patients and monkeys with amygdala lesions and intact visual cortex (Anderson and Phelps, 2001; Vuilleumier et al., 2004; Hadj-Bouziane et al., 2012). Projections from amygdala to auditory cortical areas (Amaral and Price, 1984; McDonald, 1998; Pitkänen, 2000; Yukie, 2002; Freese and Amaral, 2009), enable amygdala to drive similar perceptual and attentional enhancements in that modality (Kumar et al., 2012; Omigie et al., 2015b). In addition, amygdala can drive persisting representational changes and specialization in sensory cortical areas (Chavez et al., 2009, 2013). Amygdala also projects to frontal cortex, including heavily to ACC, BA 45 in VLPFC and anterior insula (Amaral and Price, 1984; Freese and Amaral, 2009; Vogt, 2009; Gerbella et al., 2014), likely enabling it to enhance cognitive function. Taken together, amygdala participation in stimulus representations and its extensive connectivity, enable amygdala representations to predictively enhance a network of functions that together likely constitute importance, a form of intangible knowledge. Moreover, this arrangement is consistent with predictive coding being a major strategy of the brain (Nadel and Hardt, 2011; Weston, 2012; Smith and Goodale, 2015; Saker et al., 2018), including of amygdala (Hernádi et al., 2015; Zangemeister et al., 2016).

Importance representations may develop through embodied cognition processes. For instance, in the hyperarousal form of PTSD, during the traumatic event (peritrauma) traumatic stimuli provoke extreme levels of arousal (hyperarousal) and related responses. The individual involuntarily replicates these intense responses on subsequently encountering trauma-related reminders; in other words, these reminders are characterized by extreme importance. Amygdala processing of peritraumatic bodily responses likely underlies these effects; in contrast, a study of identical twins discordant for PTSD, found genetic influences to be non-significant (Koenigs et al., 2008; Gilbertson et al., 2010; Weston, 2014). PTSD is at the extreme of the stress continuum (Ruscio et al., 2002; Forbes et al., 2005; Broman-Fulks et al., 2006), so the above processes may generalize, but this issue awaits investigation.

### Exclusiveness Representation

A very different experimental paradigm involved ewes; these animals normally form a specific memory of the scent of their own newborn offspring in the 2 h after birth, and they subsequently suckle their own offspring only while aggressively rejecting all others. Transient inactivation by lidocaine over the critical period of the cortical or medial amygdaloid nuclei resulted in indiscriminate suckling, although olfactory perception and memory retrieval were unimpaired (Keller et al., 2004). In addition, inactivation of the ewe’s olfactory system has been found to disrupt selective nursing behavior (Lévy et al., 1995; Ferreira et al., 2000). It is hypothesized that in the Keller et al. (2004) inactivation study, the ewes perceived the scent of their own offspring but failed to regard it as “exclusive” or “special,” which is likely mediated by the cortical or medial amygdaloid nuclei. Related modulatory findings mediated by rodent amygdala have been reported (Demas et al., 1997; Petrulis, 2009; Gur et al., 2014). Thus, amygdala likely mediates this further form of intangible knowledge, and environment, body and brain collaborate in ewes to produce specific adaptive knowledge, as proposed by embodied cognition.

### Ingroup Membership Representation

Cooperation among conspecifics is widespread among animals, and is strongly developed in human. It is a factor that is similarly or more important than competition-related factors such as dominance, in the achievement of health and reproductive success in human and other primates (Platt et al., 2016; Schmelz and Call, 2016; Hare, 2017). Humans rapidly form social groups, and simply belonging to a group (e.g., as a result of random allocation in experiments) leads to richer and more complex representations and amplified processing of ingroup members. For instance, belonging to a group leads to more detailed and individuated perceptual processing, greater liking and amplified processing, of ingroup members (Van Bavel et al., 2008, 2011; Cikara and Van Bavel, 2014; Guassi Moreira et al., 2017). The network that mediates the representation and amplified processing of ingroup members has been found by fMRI neuroimaging to involve amygdala, OFC, FG and striatum (Van Bavel et al., 2008, 2011; Cikara and Van Bavel, 2014; Guassi Moreira et al., 2017). Thus, amygdala participates in ingroup representation, a form of intangible knowledge, although further work is needed to more precisely specify its contribution.

### Social Status Representation

A number of studies have examined the formation and encoding of social status representations, in monkey and human. In a study of 25 group-living macaque monkeys, status relationships were measured through observation and recording of the behavioral interactions between animals. The animals’ neural structure and function were also measured by structural and functional MRI, respectively. The findings were that social status was positively related to gray matter (GM) volume of amygdala, hypothalamus, brainstem subareas and mid STS all bilaterally, as well as hippocampus and anterior DLPFC unilaterally. Social status was negatively related to GM volume of basal ganglia subregions and dorsal septum bilaterally. In addition, findings were reported of resting functional coupling between amygdala and hypothalamus, as well as amygdala and brainstem subregions, that were significantly associated with status (Noonan et al., 2014).

In two human neuroimaging studies, during training sessions a set of human faces were presented, and subjects were required to learn by trial and error the individuals’ status within the linear social hierarchy. Subjects were tested during testing sessions interleaved with training sessions, and were required to indicate an individual’s status, and give a confidence rating in their answer. The findings of functional neuroimaging were again that a network of brain regions generates and encodes social status representations, principally bilateral amygdala, bilateral anterior hippocampus, posterior hippocampus, ventromedial prefrontal cortex (VMPFC; BAs 24, 32, 9), and FG. Further, structural neuroimaging revealed that variation in subjects’ performance was significantly predicted by variation in GM volume, only of amygdala bilaterally (Kumaran et al., 2012, 2016). Moreover, the brain regions summarized above have been reported across diverse paradigms, that have investigated the formation and encoding of social status representations (see for review, Watanabe and Yamamoto, 2015). In addition, the contributions of regions within the network are being clarified. Specifically, Bayesian modeling of behavioral performance and its correlation with neural activation, suggests that VMPFC (BAs 24, 32, 9) forms and updates particular social status representations, and relays these to amygdala and hippocampus. Amygdala likely represents social status knowledge, which is also activated automatically. Posterior hippocampus likely represents linear hierarchies more generally (Kumaran et al., 2012, 2016). Taken together, there is robust evidence from diverse paradigms in monkey and man, that amygdala is a principal region in the representation of knowledge of social status, a further form of intangible knowledge. Moreover, status is learned through interactions with other individuals in a hierarchy.

### Popularity Representation

Individuals vary in popularity, which is represented by a small network that includes amygdala. The popularity of a member of a group can be quantified, by combining the ratings of liking given by fellow group members for that individual. In a study of the neural representation of popularity, members of two real-world voluntary groups of 13 members each, completed questionnaires requiring ratings of liking of other group members, and other variables, and had their faces photographed. During fMRI neuroimaging, each group member viewed the faces of group members and performed a simple cover task. The findings were that popularity was represented by a small network, comprising amygdala, VMPFC and ventral striatum. These findings remained after controlling for potential confounds, such as facial attractiveness, sex and interpersonal closeness; additionally, brain regions’ identities were determined with independent functional localizer tasks performed in the same scanning session (Zerubavel et al., 2015). Thus, amygdala participates in the representation of popularity, another form of intangible knowledge. Replications of this finding are desirable, as are findings from additional research paradigms (e.g., neuropsychology, single-cell recordings), as well as more precise specification of the contribution of amygdala to popularity representation.

### Trustworthiness Representation

Amygdala is a major part of the network that mediates trustworthiness, more specifically untrustworthiness, and this is supported by neuroimaging and neuropsychological findings. In a meta-analysis of PET and fMRI neuroimaging studies of trustworthiness represented in facial stimuli, it was found that reducing levels of trustworthiness (i.e., untrustworthiness) were associated with consistent activation of amygdala, as well as of anterior insula, VLPFC, inferior frontal gyrus (IFG) and part of the basal ganglia (Mende-Siedlecki et al., 2013). A subsequent meta-analysis of fMRI studies of trustworthiness represented in facial stimuli, again found amygdala involvement in untrustworthiness representation (Santos et al., 2016). In such studies, however, potential confounding variables such as inadvertent processing of facial attractiveness, may be affecting the findings (Mende-Siedlecki et al., 2013).

Neuropsychological approaches have examined patients with amygdala lesions, mostly brought about by disease or surgical excisions to treat epilepsy. In a study of three patients with bilateral amygdala lesions, varied facial stimuli of unfamiliar persons were presented, and the findings were that the patients were significantly impaired in trustworthiness ratings relative to neuropsychological and normal controls (Adolphs et al., 1998). A larger neuropsychological study used a group of 32 patients with unilateral amygdala lesions, a brain damage control group with focal brain lesions of any region other than amygdala, insula, or VMPFC, and a healthy control group (Koscik and Tranel, 2011). The task did not involve facial stimuli but playing the Trust Game, which entailed participating in 40 rounds of monetary exchanges with a computer player. Variables included the money given by the computer player, and the subjects’ monetary responses; these and the sums accumulated by the players were displayed to subjects. The findings were that whereas the healthy control group engaged in a tit for tat exchange strategy, the amygdala lesion patients responded to decreased or unchanged sums of money from the computer player with increased sums. These findings were interpreted as a failure to generate and represent appropriate distrust during interactions with the Trust Game player, and were associated with amygdala damage (Koscik and Tranel, 2011). More generally, behavioral studies have found that trust is likely learned through experiences, rather than being a prespecified disposition (Glanville and Paxton, 2007). In sum, substantive evidence supports the hypothesis that amygdala mediates untrustworthiness, a form of intangible knowledge. Moreover, the Koscik and Tranel (2011) findings suggest that amygdala may generate through experience this form of intangible knowledge.

### Moral Representations

Moral knowledge and judgments are a further form of intangible representation in which amygdala participates. An fMRI neuroimaging study required subjects to estimate how much money was contained in pictured transparent jars that were partly filled with penny coins across a series of trials. Subjects were incentivized by a reward structure to give inaccurate estimates (overestimates or underestimates), that in different experimental conditions benefited or harmed themselves or an associate. The behavioral findings were that self-benefitting dishonesty measures escalated across trials, consistent with reports that minor transgressions can snowball into major ones. The neuroimaging findings were that dishonesty that is self-serving and weighted by position in the succession of trials, was significantly associated with reducing activity of amygdala and anterior insula. In addition, reducing activation of amygdala across two trials in response to dishonesty, was found to predict the escalation in self-serving dishonesty on the following trial (Garrett et al., 2016). Thus, amygdala may encode some form of moral standards, whose weakening permits escalating dishonesty. Two meta-analyses have examined brain activations engaged by moral tasks. Both found consistent and reliable involvement of a network of areas, that included amygdala, OFC, MPFC, TPJ and precuneus (Bzdok et al., 2012; Eres et al., 2018). The contribution of each region in the network requires to be specified. In sum, amygdala reliably participates in the network that represents moral knowledge, another form of intangible knowledge, and some insight into amygdala’s contribution is offered by the Garrett et al.’s (2016) study.

### Further Observations and Summary

A number of intangible knowledge representations in which amygdala participates, have now been summarized. These findings have accumulated through some perceptive insights (e.g., Adolphs et al., 1998; Ewbank et al., 2009; Kumaran et al., 2012), rather than through deliberate focus on the domain of intangible knowledge representation. Additional amygdala intangible representations are likely to be revealed. In a physiological study, a rich range of visual object images was presented to monkeys who passively viewed them, while responses of a large sample of amygdala neurons were recorded. The findings were that similar proportions of amygdala neurons responded differentially to monkey faces, human faces, or most other objects. Moreover, many amygdala neurons responded quite differently to visually similar stimuli (Gothard et al., 2007), which suggests the neurons were processing features that have yet to be identified.

It has been hypothesized that amygdala’s principal representations may lie on a continuum of negative to positive valence, or “good to bad” (e.g., Paton et al., 2006; Koelsch and Skouras, 2014; Janak and Tye, 2015). It is suggested instead that amygdala may be better conceived as mediating a diversity of independent representations. That amygdala-mediated intangible knowledge representations are frequently independent, is supported by findings that social status and trustworthiness were not significantly correlated (Kumaran et al., 2012); social status was independent from social network size (Noonan et al., 2014); the impact feature representation was found after controlling for valence, arousal and other features (Ewbank et al., 2009); and amygdala neuronal activity encoding the economic value and length of plans differed substantially from that encoding reward (Hernádi et al., 2015; Zangemeister et al., 2016). Neuroanatomically, amygdala receives neural inputs that include those concerning visual objects, visual motion, audition, taste, the fattiness and grittiness of foods, temperature of foods, bodily temperature, other viscerosensations, blood acidity changes, epinephrine levels, cortisol levels, nociception and so forth (see earlier). Such wide diversity of inputs is suggestive of diverse representations being mediated by amygdala. In addition, the especially heavy and reciprocal interconnections of amygdala and PRC, which integrates fine-grained representations, are consistent with amygdala relaying a multitude of fine-grained representations to PRC; if only few coarse-grained representations needed to be processed, they would be unlikely to require such heavy and particular neural resources. Thus, this evidence supports amygdala mediation of diverse representations, but it does not resolve the issue. Hence, more direct and systematic tests are needed to definitively settle this issue, as set out in the penultimate section.

The diversity of amygdala representations likely contributes to adaptation. Dubos conceived the essence of adaptation as follows:

The real measure of health is not the utopian absence of all disease but the ability to function effectively within a given environment. And since the environment keeps changing, good health is a process of continuous adaptation to the myriad microbes, irritants, pressures and problems that daily challenge man (Dubos, 1990, p. 95).

That is, environments are highly complex, variable, and unforeseeable and individuals must continually achieve specific and tailored adaptation to the particular conditions in which they live. Amygdala’s potential for diverse and fine-grained representations can answer such demands. In addition, it is optimal for such representations to be elaborated through experience of the particular environment rather than to be prespecified in the genome. In other words, initially domain-general encoding may be elaborated through interactions with the environment into domain-specific and fine-grained representations that are tailored to the particular conditions. For example, amygdala receives nociceptive system projections, which may be regarded as domain-general. Through interactions with biting insects, which involve body, environment and brain as envisaged by embodied cognition, representations that specific insects are noxious are generated (Kavaliers et al., 1999). This elaboration of fine-grained representations is consistent with the proposal of Spunt and Adolphs (2017) that domain-general systems may through their structural connectivity, inputs and intrinsic computations, become domain-specific. Amygdala representations may also be computed by other means, such as in other brain regions that relay them to amygdala. This overall hypothesis is also consistent with findings that human cortical organization is “relaxed.” That is, in human there is high genetic control of brain size, but lower genetic control of cortical organization, particularly of high-level cortical areas, which enables increased levels of plasticity and neural circuit specialization (Gómez-Robles et al., 2015). A contrary view, however, holds that amygdala circuits may involve strong genetic prespecification (Gore et al., 2015; Beyeler et al., 2016). Currently, the formation of amygdala representations is poorly understood, so empirical studies are needed to test between these hypotheses.

In summary, the novel hypothesis is presented that a principal function of amygdala is the mediation of diverse, independent and fine-grained intangible knowledge representations, which are integrated into multimodal, meaningful and fine-grained distributed knowledge representations. Such amygdala representations encompass non-social knowledge (e.g., valence, noxiousness, economic value) and social knowledge (e.g., social status, trustworthiness, popularity). The hypothesis has considerable explanatory potential with regard to unanswered questions in several domains, as demonstrated in the next section.

## Significance for Social Processing; Clinical Significance

### Significance for Social Processing

Amygdala participates in social processing, and there is convergent evidence for this. In human and other primates, structural studies have found the volume of amygdala and other brain regions to be significantly and positively associated with the size and complexity of social networks (Barton and Aggleton, 2000; Bickart et al., 2011; Sallet et al., 2011; Von Der Heide et al., 2014). Moreover, in Sallet et al. (2011), social group size had been independently imposed on the monkey subjects, hence social network size likely drove this amygdala volume increase. Increased brain region volumes that were also associated with social network size included those of several temporal areas, anterior PFC and ACC (Sallet et al., 2011; Von Der Heide et al., 2014). Functional neuroimaging and electrophysiological studies of social tasks have also reported consistent amygdala activation, hence its inclusion in the “social brain” (Atkinson and Adolphs, 2011; Ku et al., 2011; Gotts et al., 2012; Von Der Heide et al., 2014; Rutishauser et al., 2015; Patriquin et al., 2016).

Amygdala, however, does not encode actual social behaviors. In experimental lesion studies, selective ibotenic acid lesions were made to amygdala bilaterally at 2 weeks after birth in macaque monkey infants, and the animals were observed and tested behaviorally over subsequent years. The findings were that infant-mother interactions and behaviors, as well as the species-typical repertoire of social behaviors, did not differ from those of controls. The young animals did, however, manifest a number of modest behavioral anomalies, such as retrieving without delay food placed adjacent to a fearful object such as a model snake, and displayed limited exploration of objects in some conditions but not in others (Prather et al., 2001; Machado and Bachevalier, 2003; Bauman and Amaral, 2011). When mature, the animals became less sociable in that they groomed others less, spent more time alone but alert, and females expressed little interest in the infants of other females. The animals also expressed more anxiety-related behaviors, and performed more self-directed stereotypies (Toscano et al., 2009; Moadab et al., 2015). Hence, amygdala does not directly encode social behaviors; instead its contribution is indirect and modulatory (Adolphs, 2010; Bauman and Amaral, 2011), but there is no consensus on the nature of it.

It is hypothesized that amygdala’s contribution is that of intangible knowledge, which differentially affects social behaviors compared to other behaviors. Specifically, conspecifics are commonly characterized in terms of being, for instance: supportive, caring, kind, loyal, faithful, important, trustworthy, interdependent, dishonest, hostile, critical, tolerant, constructive, understanding, appreciative, respectful, contemptuous, fair, dominant, subordinate, interesting, close, special, rich, aggressive and so forth. Moreover, many of these features have major roles in social relationships (e.g., Wright, 1985; Reynolds and Mansfield, 1999; Gilligan, 2000; Rachman, 2010; Lavner and Bradbury, 2012; Platt et al., 2016; Fincham and May, 2017). In contrast, other objects and stimuli are infrequently so characterized. That is, intangible knowledge processing is more extensive, critical, and finer-grained in social contexts. Amygdala processes some of these features, which may explain its influence on social processing, and its expansion with larger and more complex social networks (Barton and Aggleton, 2000; Bickart et al., 2011; Sallet et al., 2011; Von Der Heide et al., 2014). Amygdala disruption, moreover, is likely to preferentially impair social cognition and social interactions, as occurs in ASD and is discussed next.

### Clinical Significance: Autism Spectrum Disorder

#### Amygdala Is Disrupted in ASD

Amygdala is commonly disrupted in ASD, and the presented amygdala hypothesis offers testable explanations of a number of prominent but unexplained ASD symptoms and features. Amygdala dysfunction in ASD has long been suggested (Bachevalier, 1994; Baron-Cohen et al., 2000) and is supported by functional evidence, which includes neuroimaging findings of amygdala functional abnormalities (see for meta-analysis, Patriquin et al., 2016), and electrophysiological findings in two ASD patients of abnormal selectivity of a subpopulation of amygdala neurons (Rutishauser et al., 2013). In addition, deep brain stimulation of basolateral amygdala but not adjacent brain regions, was found to ameliorate numerous ASD symptoms, as well as self-injurious behaviors (Sturm et al., 2013). Structural evidence includes structural neuroimaging findings of reduced amygdala volume in adolescents and adults (Stanfield et al., 2008; Schumann and Amaral, 2009; Via et al., 2011; Sato et al., 2017b), cellular findings of reduced amygdala neuron numbers (Schumann and Amaral, 2006) or amygdala neuron density (Wegiel et al., 2014), and findings of an abnormal amygdala growth trajectory that is also associated with ASD symptomatology (Munson et al., 2006; Nacewicz et al., 2006; Stanfield et al., 2008; Schumann and Amaral, 2009; Schumann et al., 2009; Kim et al., 2010). Together, these provide robust and convergent evidence of amygdala disruption in ASD. Such disruption can likely explain the following ASD symptoms and features.

#### Disorganized Scanpaths and Apparent Social Disinterest

A core feature that manifests early, persists throughout life, is independent of IQ, and whose mechanism is unclear (Frazier et al., 2017), is the disorganized visual scanpaths of ASD individuals. Specifically, eye-tracking recordings of TD controls’ scanpaths of scenes reveal that such scanpaths are organized and selective. Stimuli such as humans, their faces, and eyes commonly receive the majority of visual processing, whereas trivial stimuli receive fleeting processing at most (Klin et al., 2002, 2003, 2009). These and other findings suggest that importance, valence, salience, arousingness, and other intangible features are major factors in the organizing of scanpaths (Niu et al., 2012; Vuilleumier, 2015; Schomaker et al., 2017). ASD individuals’ visual scanning of scenes, in contrast, is atypical: it is unorganized, unselective, and almost random (e.g., Klin et al., 2002, 2003, 2009; see for meta-analysis, Frazier et al., 2017). ASD individuals’ object recognition and face recognition, however, are essentially intact (Jemel et al., 2006; Simmons et al., 2009; Weigelt et al., 2012). When orderly scanpaths in ASD have been reported, moreover, they were found to be driven not by intangible knowledge as in TD individuals, but by physical aspects of the scene, namely audio-visual synchronies (Klin et al., 2009). A subsequent fine-grained study examined ASD and TD individuals’ visual scanpaths that were executed while viewing complex naturalistic images. It was found that ASD differences in scanpaths did not emerge early at the basic visual or object levels, but emerged later in the time course of viewing and particularly at the level of processing of meaning. Moreover, fixations to social stimuli were delayed, whereas fixations to artifactual stimuli were speeded (Wang et al., 2015). Taken together, it is hypothesized that deficits of intangible knowledge are sufficient to induce the commonly unorganized visual scanpaths in ASD. Moreover, amygdala mediates intangible knowledge, and it participates in the organizing of scanpaths (Gonzalez Andino and Grave de Peralta Menendez, 2012), hence its disruption may underlie ASD scanpath disorganization.

Auditory stimuli such as parents’ voices, the individual’s own name being called, and language sounds, commonly elicit interest in TD children. ASD children, in contrast, are frequently unresponsive to their parents’ voices, to their own name being called, and to language sounds, according to observational and experimental evidence (Kanner, 1943; Klin, 1991; Jolliffe et al., 1992; Nadig et al., 2007; Miller et al., 2017). Basic sensory function, however, is found by audiometric tests to be normal in the great majority of ASD individuals (Tharpe et al., 2006; Tas et al., 2007). Similarly in the visual modality, ASD individuals are commonly reported to be apparently uninterested in fellow humans, including family members, peers, acquaintances, and social relationships generally, despite intact face recognition (American Psychiatric Association, 2013; Kanner, 1943; Jolliffe et al., 1992; Simmons et al., 2009; Weigelt et al., 2012).

At the neural level, TD individuals show enhanced high-level auditory cortex processing of human speech sounds; in contrast, ASD individuals commonly fail to show such enhanced high-level cortical processing. Nevertheless, corresponding processing of acoustically and complexity matched control sounds does not differ significantly between ASD and TD groups as measured by fMRI or event-related brain potentials (Ceponiene et al., 2003; Gervais et al., 2004; Lepistö et al., 2005; Whitehouse and Bishop, 2008). Similarly in the visual modality, high-level visual area FG has the capacity for normal levels of activation, but is commonly hypoactive when performing social tasks, and this has been ascribed to impaired modulation (Grelotti et al., 2005; Pierce and Redcay, 2008; Perlman et al., 2011; Patriquin et al., 2016). Together, these findings suggest there is a failure of enhancement of otherwise intact auditory and visual cortical processing in ASD individuals. This is suggested to manifest as apparent indifference to parents, peers, own name being called, speech, and so forth. Amygdala disruption likely participates in such impaired enhancement. Amygdala is heavily and extensively interconnected with visual cortex, and has an excitatory effect on it (Freese and Amaral, 2005, 2006, 2009; Smith et al., 2009). Further, in a monkey lesion study, facial expressions were found to enhance visual cortical activity in control animals, but such enhancement was abolished in monkeys with amygdala lesions (Hadj-Bouziane et al., 2012). Moreover in ASD, connectivity between amygdala and FG is reduced, and the latter is commonly hypoactive (Conturo et al., 2008; Kleinhans et al., 2008; Patriquin et al., 2016). Taken together, further studies are required, but current evidence is consistent with the hypothesis that apparent disinterest in visual social stimuli in ASD, likely arises from impaired enhancement of activity in high-level visual cortex, which is likely driven by amygdala disruption (see Kleinhans et al., 2011; Hadj-Bouziane et al., 2012). Corresponding processes in high-level auditory cortex, which is also hypoactive in ASD (see above), and is interconnected with amygdala (McDonald, 1998; Yukie, 2002; Freese and Amaral, 2009), are plausible and require testing.

A further marked feature is the inability to understand social situations reported by ASD individuals (Jolliffe et al., 1992; Williams, 1996; Grandin, 2005). Given their intact physical knowledge but impairments at the meaning level (see above), it is hypothesized that impaired intangible knowledge likely contributes. Furthermore, this impairment in understanding has been reported as a major source of the stress and dysphoric emotions that are a common feature of ASD (Jolliffe et al., 1992; Williams, 1996; Grandin, 2005).

#### Preference for Concrete Cognition

Amygdala dysfunction may explain the preference for concrete and mechanical cognition in ASD, a feature which is evidenced by multiple findings (Baron-Cohen and Wheelwright, 1999; Klin and Jones, 2006; Klin et al., 2007, 2009; Ropar and Peebles, 2007; Wang et al., 2015). Generally, an impaired brain system can facilitate enhanced use and development of brain systems remaining intact, and this has been reported for diverse brain diseases that affected disparate brain regions (Kapur, 1996; Miller et al., 1998, 2000; Thomas-Anterion et al., 2010; Schott, 2012; Midorikawa and Kawamura, 2015). For example, anterior temporal lobe degeneration, which manifests in social and language impairments, can co-occur with posterior parieto-occipital enhancement, which is associated with outstanding artistic development (Miller et al., 2000; Schott, 2012; Midorikawa and Kawamura, 2015). Correspondingly in ASD, impaired neural systems for intangible cognition are suggested to facilitate enhanced use and development of preserved systems for concrete and mechanical cognition. Hence, the strongly developed and prominent concrete and mechanical cognition that is frequently reported in ASD.

#### Heterogeneity of ASD

Heterogeneity characterizes most medical disorders (Lawrie, 2017), but it is particularly severe in ASD (Schumann et al., 2011), and impedes progress in understanding of the mechanisms of the disorder. Amygdala is commonly regarded as mediating a homogeneous class of representations such as valence or fear (see earlier), rendering the heterogeneity of ASD difficult to comprehend. Amygdala, however, likely mediates a diversity of independent representations as argued above, rendering ASD heterogeneity more intelligible. For example, inputs to amygdala, which is one of the most widely interconnected of brain regions (Young et al., 1994), include visual, oral somatosensory, nociceptive, blood acidity and stress hormone inputs, which likely participate in different amygdala representations. Hence, for the richly interconnected amygdala, disrupted inputs that vary in number and in combinations, will elicit unusually heterogeneous patterns of impairments. Moreover, any one dysfunction is unlikely to be universal in ASD (see Rapin, 2006). Thus, amygdala’s diversity of connectivity and functions is likely a substantive contributor to ASD heterogeneity.

#### Activities of Daily Living

Impaired activities of daily living in ASD are widespread, disproportionate to IQ, particularly disabling, stressful both for the affected individuals and their carers, and the mechanisms are unclear (Green et al., 2000; Howlin et al., 2013; Duncan and Bishop, 2015). It is likely that an inability to plan underlies impairments in some routine activities, such as making phone calls, handling money and using public transport (Green et al., 2000). Amygdala participates in planning processes (Hernádi et al., 2015; Zangemeister et al., 2016), hence it is hypothesized that amygdala dysfunction may participate in the impairment of such daily living activities. In addition, impaired trustworthiness representations likely contribute to social naivety and vulnerability, and impaired noxiousness representations to an inability to appreciate the dangerousness of objects and situations (see American Psychiatric Association, 1994, 2013; Wing, 1976), which are further features that impair daily living in ASD. In summary, the presented amygdala hypothesis offers testable explanations of a number of prominent but poorly understood symptoms and features of ASD. A comprehensive account of ASD symptomatology, however, will involve dysfunctions of additional brain regions.

## Cautions and Directions for Future Research

The generation and representation of intangible knowledge has been little researched, and more extensive studies of its contributions to cognition, and of amygdala’s contributions are needed. Diverse studies have examined the representation of trust in the brain, and similar research attention should focus on further forms of intangible knowledge, which have often been investigated by few studies. The forms of intangible knowledge that are important in social relationships and social living, need to be delineated and merit more research attention.

Almost all sensory systems relay strongly to amygdala, predominantly to the lateral and basal nuclei, and these same nuclei relay heavily and reciprocally to PRC, as summarized earlier. This pattern of connectivity suggests the existence of significant neural circuits but these have not been examined. Their existence may be tested by means of diffusion tensor imaging in human, or by transneuronal viral tracers in monkey. The structure of these pathways suggests, interactive, recurrent processing, which is engaged in the formation of fine-grained, meaningful representations (see earlier). Such processing has been demonstrated in the vision—amygdala pathway (Sato et al., 2017a); the prediction of such processing in the amygdala—PRC pathway could be tested with intracranial electroencephalography in suitable but rare human patients, or single-cell recording in monkey.

The hypothesized diversity of amygdala-mediated representations raises a number of questions. Is the diversity real or an artifact of the experimental paradigms? There is some evidence that various amygdala representations are independent (e.g., Ewbank et al., 2009; Kumaran et al., 2012), but it is not sufficient. Future investigations of a particular amygdala representation should also examine potentially overlapping constructs, so as to be able to test systematically and explicitly, amygdala representations in terms of distinctiveness, fine-grained nature, or independance from fear, valence, and so forth. How does the diversity come about? It is likely that differing inputs may partly account for different representations. For example, pain, stress hormone, and gustatory inputs to amygdala are suggested to be processed to generate corresponding representations, such as noxiousness, importance, and valence, respectively. A useful strategy would be to examine disruptions of particular sensory systems or other inputs to amygdala, and their associations with particular forms of intangible knowledge impairment, as was illustrated by Kavaliers et al. (1999). In addition, replications of the Kavaliers et al.’s (1999) study are desirable, and verification needed that amygdala is indeed crucially involved in this processing. Amygdala receives high-level visual and auditory inputs, engages in markedly lengthy processing of them, and generates representations that differ from those of the sensory inputs (Wang et al., 2014; Omigie et al., 2015a; Minxha et al., 2017). The nature of the amygdaloid computations performed, and how the amygdaloid inputs and outputs differ, should further illuminate representation formation. How can the proposed representational diversity be encoded? The challenge of encoding a rich diversity of representations in a common region of brain tissue, has been answered in other brain regions, such as VTC. The latter encodes an infinite variety of face and other object forms by means of a powerful coding strategy, population coding. That is, the forms of diverse stimuli are encoded by means of different patterns of activation across a common region of cortex (Rolls, 2000; Haxby et al., 2004). The operation of population coding in monkey amygdala has been reported by Rolls (2000), and requires more detailed investigation.

In ASD, impaired intangible knowledge is hypothesized to underlie a number of symptoms and features, and empirical testing is required. Such impairment of intangible knowledge can be tested with the property-listing paradigm and its variants, in which the participants’ task is to list all the features they can for each presented item, or to rate the presence or influence of given features in each item (e.g., Taylor et al., 2007; Gainotti, 2012). Open-ended questions such as “What kind of person is Elizabeth?” (see Klin et al., 2007) may also be revealing about intangible knowledge deficits. To further examine the atypicality of visual scanpaths in ASD, the paradigm used by Gonzalez Andino and Grave de Peralta Menendez (2012), could be adapted for use with ASD and control presurgical patients, or eye tracking studies may be combined with investigation of prominent fixation regions in terms of salience, valence, and other intangible features. Impaired intangible knowledge is hypothesized to drive the preference for concrete cognition. An empirical prediction is that independent measures of abnormalities of intangible cognition and of concrete cognition should be inversely associated. Amygdala lesions in monkeys abolished the normal enhancement of visual cortex to emotional facial expressions (Hadj-Bouziane et al., 2012). It would be valuable to explore whether a corresponding impairment occurs in the auditory modality too, as this is a candidate mechanism for the apparent indifference to parents’ voices, to own name being called, and to speech sounds in ASD. More generally, studies of sensory systems, amygdala, and PRC, and their involvement in intangible knowledge generation and representation, may facilitate advances in embodied cognition theory (see Varela et al., 1991; Chiel and Beer, 1997; Clark, 1999, 2008).

## Conclusion

A hypothesis of an amygdala function that builds on and extends earlier proposals has now been presented, and makes several novel and substantial contributions to the literature. The hypothesis highlights the domain of intangible knowledge, which is an influential factor in social and other behaviors, but which has been little researched. The hypothesis elucidates the function that engages the likely heaviest of amygdala interconnections, and proposes it as a principal amygdala function. It has not received proportionate research attention. The hypothesis proposes that this amygdala function is the mediation of diverse, independent, meaningful, and fine-grained intangible knowledge representations; this differs from the few coarse-grained representations of relevance, salience, and so forth that are typically proposed. The formation of intangible knowledge representations and amygdala representations is not well understood. Evidence and hypotheses are set out on the formation of some of these representations. Amygdala’s contribution to social cognition is unclear, following the monkey neonatal lesion experiments that refuted the proposal that amygdala mediates actual species-typical social behaviors (Bauman and Amaral, 2011). A novel answer to this open and important question is presented. On the basis of the main hypothesis, novel hypotheses are presented to explain several core ASD symptoms, which are currently poorly understood, together with clear and specific proposals for empirical tests. This demonstrates the explanatory potential and broad significance of the main hypothesis. Taken together, the presented hypothesis should progress understanding of amygdala generally, of embodied cognition processes, of social processing, of clinical disorders with amygdala involvement, and is relevant for a comprehensive account of amygdala function.

## Author Contributions

The author confirms being the sole contributor of this work and approved it for publication.

## Conflict of Interest Statement

The author declares that the research was conducted in the absence of any commercial or financial relationships that could be construed as a potential conflict of interest.
